# The Niagara Meeting

**Published:** 1886-07

**Authors:** 


### THE NIAGARA MEETING.

The present indications point to an unusually large meeting of the American Dental Association. Niagara was the birthplace of the Society, and it has always been a favorite point for the meetings. This year the natural attractions are greatly increased from the purchase by the State of the lands surrounding this wonderful cataract, and its conversion into a free park. The visitor can now go where he will, without having a demand made upon him for entrance money.

During the past winter a fine building has been erected for a hall and theatre, and this has been engaged for the meeting. All the hotels, except the Cataract House (which has been shorn of its principal attractions), have offered reduced rates to the delegates, and as there is room enough, it is expected that there will be a very large representation. It has been the habit of many to attend one or two days—long enough to be able to boast that they had been in attendence upon the National Meeting—and then quietly to disappear. This is no way to obtain any of the benefits of the meeting. It requires constant attendance and a close watching of the proceedings to catch the spirit of the meeting, but to one who does this there is nothing which he will find so professionally profitable. There will be plenty of time for recreation between the sessions, and arrangements are being made by the local committee for excursions and rides to all the points of interest, at reduced rates.

There is no place in America that is so restful as Niagara. It is to be hoped that many will come a few days in advance, and thus be fully prepared for the meeting, and that more yet will stay for a time after the meeting has closed. The social interest of the occasion would be much enhanced if members would bring their wives and daughters with them. They often need rest and change quite as much as the head of the family. Bring them to Niagara this summer.

